# Impact of Long-Term Non-Communicable Diseases on SARS-COV-2 Hospitalized Patients Supported by Radiological Imaging in Southern Pakistan

**DOI:** 10.7759/cureus.67110

**Published:** 2024-08-18

**Authors:** Ali Qureshi, Syed Azhar Syed Sulaiman, Pushp Lata Rajpoot, Maryam Mohammed Sahli, Narendar Kumar, Shireen Bhurgri, Nur Aizati Athirah Daud

**Affiliations:** 1 Discipline of Clinical Pharmacy, School of Pharmaceutical Sciences, Universiti Sains Malaysia, Penang, MYS; 2 Department of Health Education and Promotion, College of Public Health Education and Tropical Medicine, Jazan University, Jazan, SAU; 3 Department of Clinical Pharmacy, University of Sindh, Jamshoro, Jamshoro, PAK; 4 College of Pharmacy, Liaquat University of Medical and Health Sciences, Jamshoro, PAK

**Keywords:** ncd, chest x-ray, mortality, roc curve, sars-cov2

## Abstract

COVID-19 patients with already existing chronic medical conditions are more likely to develop severe complications and, ultimately, a higher risk of mortality. This study analyzes the impacts of pre-existing chronic illnesses such as diabetes (DM), hypertension, and cardiovascular diseases (CVDs) on COVID-19 cases by using radiological chest imaging. The data of laboratory-confirmed COVID-19-infected hospitalized patients were analyzed from March 2020 to December 2020. Chest X-ray images were included to further identify the differences in X-ray patterns of patients with co-morbid conditions and without any co-morbidity. The Pearson chi-square test checks the significance of the association between co-morbidities and mortality. The magnitude and dimension of the association were calibrated by the odds ratio (OR) at a 95% confidence interval (95% CI) over the patients’ status (mortality and discharged cases). A univariate binary logistic regression model was applied to examine the impact of co-morbidities on death cases independently. A multivariate binary logistic regression model was applied for the adjusted effects of possible confounders. For the sensitivity analysis of the model, receiver operating characteristic (ROC) was applied. Patients with different comorbidities, including diabetes (OR = 33.4, 95% CI: 20.31-54.78, p < 0.001), cardiovascular conditions (OR = 24.14, 95% CI: 10.18-57.73, p < 0.001), and hypertension (OR = 16.9, 95% CI: 10.20-27.33, p < 0.001), showed strong and significant associations. The opacities present in various zones of the lungs clearly show that COVID-19 patients with chronic illnesses such as diabetes, hypertension, cardiovascular disease, and obesity experience significantly worse outcomes, as evidenced by chest X-rays showing increased pneumonia and deterioration. Therefore, stringent precautions and a global public health campaign are crucial to reducing mortality in these high-risk groups.

## Introduction

Coronavirus (COVID-19), caused by syndrome-coronavirus-2 (SARS-CoV-2) is a horrible pandemic global disease that was initially raised in the city of Wuhan, China, and spread in a serious pandemic manner, making its way all over the world [1]. As the novel COVID-19 continues to spread, there are still many limitations in the knowledge of researchers about this virus, which exactly this virus would impact critically. The COVID-19 infection ranges from the common cold to fatal respiratory tract infections. Patients with COVID-19-induced pneumonia generally require intensive care during hospitalization, which mostly leads to respiratory collapse and eventually death [2,3].

Previously, in severe acute respiratory syndrome and Middle East respiratory syndrome, it was reported that the population with already existing chronic medical conditions was more likely to develop severe complications. Ultimately, the mortality rate was higher among patients with chronic illnesses [4]. Previous studies reported that COVID-19-infected patients, along with those with comorbid conditions, may have a poor prognosis [5]. Several retrospective studies of elderly and middle-aged patients with novel COVID-19 found that the elderly population with comorbid conditions is more susceptible to severe illness and is two- to five-fold more likely to be admitted to the ICU, augmenting the two-fold increased risk of mortality.

Studies have shown that diabetes can increase the susceptibility to various infections, including respiratory tract infections [5,6]. Similarly, a poor prognosis and increased deaths due to pneumonia have been evidenced in diabetic cases. The main reason behind this risk of infection is the compromised immune mechanism [7,8]. Moreover, few studies recommend optimizing blood glucose levels along with rational treatment modalities to reduce the risk of COVID-19-related complications [9,10]. It has been estimated that patients with pre-existing medical conditions have significantly fewer chances to survive as compared to those without any comorbidities [11].

Hypertensive cases that have been diagnosed with COVID-19 are in great peril due to COVID-19. The studies also confirm that the patients with severe infection were more likely to undergo ICU admission or even worse consequences can be seen among the hypertensive patients [12,13]. Those complications can cause myocardial injury and ultimately death [14-16]. Among elderly individuals, the risk of mortality from COVID-19 is higher if they have a history of any medical condition such as coronary artery disease (CAD), hypertension, or heart failure (HF) [11]. Therefore, the present study aimed to analyze the impact of non-communicable, long-term medical conditions, such as diabetes (DM), hypertension, cardiovascular diseases (CVDs), and obesity, in COVID-19 patients, supported by radiological imaging in southern Pakistan.

## Materials and methods

Study design and patient sampling

A retrospective, single-center observational study was performed at a tertiary care hospital located in Hyderabad, Pakistan. Patient data were extracted from patients’ profiles with their consent in a retrospective manner. Profiles of patients below 18 years of age and profiles without PCR were excluded from this study. Unclear profiles along with data from unconfirmed screened patients were also not included in the present study.

The profiles of COVID-19-infected patients were assessed and recorded, including various parameters such as the COVID-19 positive test report and the presence of any co-morbid conditions during the period of March 2020 to December 2020. A convenient sampling technique was chosen to collect the COVID-19-infected patient’s data.

​​​Inclusion criteria****

Patients’ profiles with positive polymerase chain reaction (PCR) results for COVID-19 were included. Only hospitalized COVID-19 patients with comorbid conditions such as diabetes, hypertension, and cardiovascular conditions were included in the study. Patients’ profiles from the high-dependency unit (HDU) and the intensive care unit (ICU) were included.

Exclusion criteria****

Profiles of patients under 18 years and profiles without PCR reports were excluded from this study. Patients with malignancies such as tumors or cancer were not part of this study. Profiles with chronic liver disease, asthma, tuberculosis, and chronic kidney disease were also excluded. Patients who were transferred to other hospitals or who were discharged on request (DOR) were excluded.

Data collection method

A proforma was developed in the English language as a tool of study using previously reported literature on COVID-19 [15,16]. The tool was designed to gather complete demographic and clinical data on the patient for analysis. The proforma was designed in three main sections, including demographic details, the presence of any co-morbidity, and COVID-19 details.

Patients’ age, gender, and locality (urban or rural) details were collected as part of demographic details. Additionally, co-morbidity details, including diabetes, hypertension, and cardiovascular histories, along with body mass index, were noted from the recorded data. In addition, random blood sugar (RBS) levels, SpO2, D-dimmer value, and pulse rate were also recorded from the COVID-19-infected patients' history.

COVID-19 details were checked to verify the patient’s status (death case or recovered case), and the COVID-19 detection test report (real-time polymerase chain reaction report) was considered mandatory. Furthermore, chest X-rays for all COVID-19 patients were taken and interpreted by an experienced cardiothoracic radiologist with over 10 years of experience in interpreting chest X-rays. Moreover, the radiologist was kept blind to the PCR results of COVID-19 patients and previously reported X-rays. The chest X-rays were interpreted on PACS (picture archive and reporting system) using diagnostic monitors.

Ethics approval

The Institutional Bioethics Committee certificate (IBC) was issued by the Office of Research Innovation & Commercialization (ORIC), University of Sindh, Jamshoro, dated 07/10/2021, under letter no. ORIC/SU/843.

Statistical analysis

The data was presented as their frequencies and percentages. For the statistical analysis, use IBM Corp. Released 2020. IBM SPSS Statistics for Windows, Version 27.0. Armonk, NY: IBM Corp. for the descriptive statistics and logistic model design, respectively. A Pearson chi-square test was performed to assess the level of significance of the obtained data. The logistic regression model was applied to check the association. The odds ratio (OR) at a 95% confidence interval (95% CI) was determined to assess the level of risk association, i.e., OR > 1 indicates that there are greater odds of the event happening in the exposed versus the non-exposed group, while OR < 1 implies the odds of the event happening in the exposed group are less than in the non-exposed group. A p-value of < 0.05 was considered statistically significant. The model sensitivity was determined through the receiver operating characteristic (ROC) curve. The value for area under the curve (AUC) above 0.8 was considered excellent, 0.6 to 0.7 was considered acceptable, and values below 0.6 and 0.5 were considered poor.

## Results

The data of 723 hospitalized COVID-19 cases was obtained. Of the total, 103 profiles were rejected, as 20 profiles belonged to patients below 18 years old, 13 with a history of chronic liver disease (CLD), 10 with chronic kidney disease (CKD), 15 with asthma, two with tuberculosis (TB), 24 were discharged on request (DOR), and 19 were transferred to other hospitals. Moreover, profiles from 20 patients had no significant evidence of COVID-19 infection, such as a negative PCR report; therefore, they were also excluded. Finally, the data of 600 patients was included for evaluation.

Among these cases, 75.8% (n = 455) were males and 24.2% (n = 145) were females (Table 1). About 77% (n = 462) of the patients came from urban settings. The mean age +SD was 41.8 + 17.5 years. Of the total sample, 30.2% (181) were mortal cases (Table 1).

**Table 1:** Summary of demographic variables in the study of COVID-19 patients (N=600)

Variables	Total N=600	Died N=181	Recovered N=419
Gender			
Male	455 (75.8%)	134 (74%)	321 (76.6%)
Female	145 (24.2%)	47 (26%)	98 (23.4%)
Locality			
Urban	462 (77%)	114 (63%)	348 (83%)
Rural	138 (23%)	67 (37%)	71 (17%)
Age Groups			
16 – 30 years	212 (35.3%)	7 (3.9%)	205 (49%)
31 – 45 years	150 (25%)	19 (10.5%)	131 (31.3%)
46 – 60 years	129 (21.5%)	62 (34.3%)	67 (16%)
61 – 75 years	88 (14.7%)	73 (40.3%)	16 (3.6%)
>76 years	21 (3.5%)	20 (11.2%)	1 (0.2%)

As stated in Table 2, patients with a history of diabetes were 33.4 times more likely to die in COVID-19 as compared to non-diabetic subjects (95% CI: 20.31-54.78, p < 0.001). Patients with cardiovascular disease were 24.14 times more likely to die (95% CI: 10.18-57.73, p < 0.001), and patients with hypertension were 16.9 times more likely to die (95% CI: 10.20-27.33, p < 0.001). This shows that different co-morbidities increase the susceptibility to death in COVID-19.

**Table 2:** Co-morbidities in COVID-19-infected patients

Co-morbidities	Total N=600	Died N=181	Recovered N=419	O.R.	95% Confidence Interval		p-value
					Lower	Upper	
Non-diabetic	442 (73.7%)	52 (28.7%)	390 (93.1%)	Ref			< 0.001
Diabetic	158 (26.3%)	129 (71.3%)	29 (6.9%)	33.4	20.31	54.78	< 0.001
Without CVDs	547 (91.2%)	134 (74%)	413 (98.6%)	Ref			< 0.001
With CVDs	53 (8.8%)	47 (26%)	6 (1.4%)	24.14	10.18	57.73	< 0.001
Non- hypertensive	479 (79.8%)	86 (47.5%)	393 (93.8%)	Ref			< 0.001
Hypertensive	121 (20.2%)	95 (52.5%)	26 (6.2%)	16.9	10.20	27.33	< 0.001

**Figure 1:**
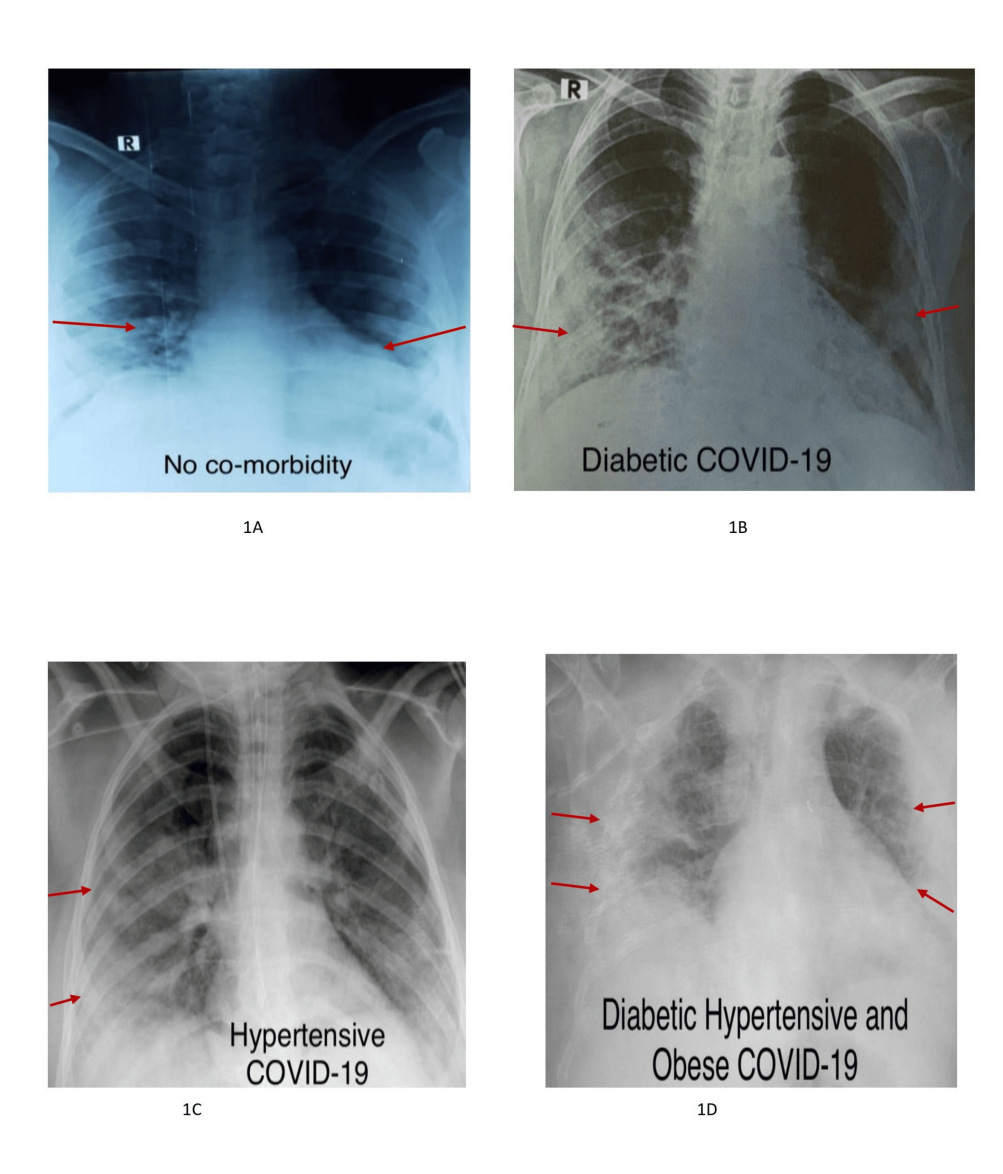
Anterior-posterior chest X-ray (CXR) imaging of COVID-19 patients Figure 1A subject without co-morbidity: anterior-posterior CXR showing bilateral symmetrical soft tissue opacities predominantly in the periphery and lower zones. Figure 1B: diabetic subject: posterior-anterior view showing consolidations in bilateral lower zones more on the right side. Figure 1C: hypertensive subject: posterior-anterior CXR showing airspace opacification in bilateral mid- and lower zones predominantly involving the right lung. The right sided CVP line, NG tube, and ETT are seen. Figure 1D: Subject with various co-morbidities together: anterior-posterior view showing poor inspiration with bilateral predominantly peripheral infiltrates more on the right side.

The anterior-posterior chest X-ray (CXR) imaging showed that COVID-19 patients without any comorbid conditions showed bilateral symmetrical soft tissue opacities predominantly in the periphery and lower zones (Figure 1A). However, patients with diabetes showed clear consolidations in bilateral lower zones, more specifically on the right lobe (Figure 1B). Hypertensive COVID-19 patients show airspace opacification in the bilateral middle and lower zones predominantly, involving the right lung (Figure 1C). Obese COVID-19 patients showed poor inspiration, with predominantly peripheral infiltrates on the right side (Figure 1D). 

Table 3 explains that overweight patients were 3.364 times more likely to die in COVID-19, whereas obese patients were 10.341 times more likely to die in COVID-19 at a 95% confidence interval (2.589-41.299) and p < 0.05, showing a strong significance of the association of obesity with mortality in COVID-19 hospitalized patients. Table 4 explains that random blood sugar levels greater than 140 mg/dl were 49.782 times more likely to cause death in COVID-19, with p < 0.001 showing a highly statistically significant association. Similarly, the likelihood of death increases with an increased pulse rate. Patients with a pulse rate < 90 beats per minute were 437.7 times more likely to die. Meanwhile, Table 5. shows the multivariate binary logistic regression model for various factors that contribute to death among COVID-19 patients. Diabetic patients showed a significant association with mortality with an adjusted odds ratio of 27.52 (95% confidence interval: 14.867-50.938, p < 0.001). Predominantly, obese patients had the highest adjusted odds ratio of 76.20 (95% confidence interval: 6.186-244.157, p < 0.001).

**Table 3:** Body Mass Index of COVID-19 infected patients

Variables	Total N=600	Died N=181	Recovered N=419	OR	95% Confidence Interval		p-value
					Upper	Lower	
Underweight	18 (3%)	11 (6.1 %)	7 (1.7%)	Ref			< 0.001
Normal	469 (78.2%)	68 (37.6%)	401 (95.7%)	0.108	0.040	0.288	< 0.001
Overweight	44 (7.3%)	37 (20.4%)	7 (1.7%)	3.364	0.968	11.684	0.056
Obese	69 (11.5%)	65 (35.9%)	4 (1%)	10.341	2.589	41.299	0.001

**Table 4:** Clinical variables (RBS, pulse rate, and SpO2 level, D-dimmer) associated with mortality in COVID-19 RBS stands for random blood sugar level (mg/dL), last recorded pulse rate (beats per minute) range was taken as standard, SpO2 level > 94% = more oxygen saturation and < 94% = less oxygen saturation, D-dimmer value > 0.5 represents that clotting was higher. X2 represents the Chi-square test value, and p < 0.05 represents the statistical significance of the association.

Variables	Died N=181(%)	Recovered N=419 (%)	X^2^	p-value
RBS (mg/dL)				
< 140	97 (18.8)	418 (81.2)	221.59	< 0.001
> 140	84 (98.8)	1 (1.2)		
Pulse Rate (bpm)				
70 – 80	6 (1.5)	404 (98.5)	514.71	< 0.001
81 – 90	45 (77.6)	13 (22.4)		
> 90	130 (98.5)	2 (1.5)		
SpO_2 _level (%)				
>94%	146 (42.6)	197 (57.4)	59.44	< 0.001
<94%	35 (13.6)	222 (86.4)		
D-dimmer (μg/mL)				
< 0.5	112 (59.9)	75 (40.1)	113.96	< 0.001
> 0.5	69 (16.7)	344 (83.3)		

**Table 5:** Multivariate logistic regression model of comorbidities adjusting for mortality DM: Diabetes mellitus, CVDs: Cardiovascular diseases, HTN: Hypertension, aOR: Adjusted odds ratio

Variables	aOR	95% Confidence Interval		p-value
		Lower	Upper	
DM	27.52	14.867	50.938	< 0.001
CVDs	8.01	2.262	28.368	0.001
HTN	6.27	2.993	13.153	< 0.001
OBESE	76.20	23.976	242.182	< 0.001

**Figure 2:**
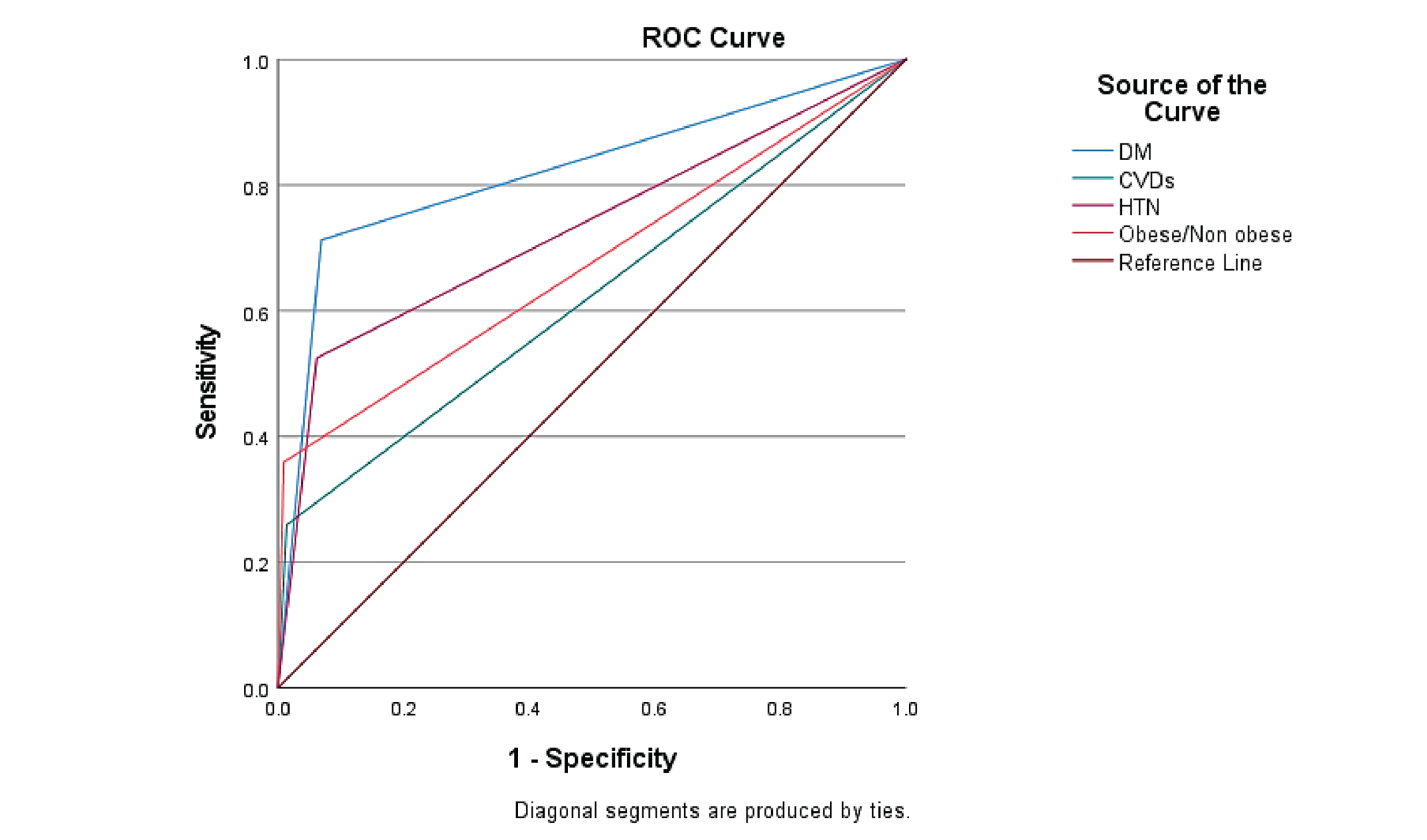
Receivers operating characteristic (ROC) curve for sensitivity and specificity of the model Sensitivity analysis of the model with diabetes (DM), cardiovascular diseases (CVDs), hypertension (HTN), and obesity with the help of the receiver operating characteristic (ROC) curve

Figure 2 demonstrates that the receiver operating characteristic (ROC) curve was applied to check the sensitivity (true positive rate) and specificity (false positive rate) of the model described in Table 5. The ROC analysis revealed that the area under the curve (AUC) for diabetic patients was 0.822 (95% confidence interval: 0.784-0.864, p < 0.001), for cardiovascular diseases it was 0.623 (95% confidence interval: 0.570-0.675, p < 0.001), for hypertension it was 0.731 (95% confidence interval: 0.683-0.780, p < 0.001), and for obesity it was 0.75 (95% confidence interval: 0.623-0.726, p < 0.001).

## Discussion

Multiple comorbid conditions are generally associated with the severity of the progression of COVID-19 disease. It was reported in previous studies that COVID-19 patients who suffer from type 2 diabetes were more likely to have serious symptoms and an increased severity of COVID-19 complications [8,17]. In a cohort study conducted on 7337 patients infected with COVID-19 with and without type 2 diabetes, it was observed that those with type 2 diabetes required more serious interventions for their hospital stay versus those who were non-diabetic [8]. Furthermore, it was reported that those with poorer control of blood glucose levels had an all-around increased mortality rate than those with better glucose control [18,19].

Many of the negative outcomes of COVID-19 management have been associated with the cardiovascular system. Hypertension among COVID-19 patients could be a drastically lethal factor, as one study confirms that the patients with severe infection among the ICU admissions observed to have the utmost need of mechanical ventilation, and also the prevalence of mortality could be an increase in cases of hypertensive condition with a percentage of 23.7% (n=173) and 15%, respectively [20,21]. According to the results of the present study, of the total 600 COVID-19 admitted cases, 20.2% (n=121) were hypertensive, and 79.8% (n=479) were non-hypertensive. Moreover, it was found that among the total n=121 hypertensive cases, 52.5% (n=95) were death cases with a p-value of < 0.001. The chest X-ray of hypertensive COVID-19 patients displays the lungs with significant, patchy opacities, reflecting more severe inflammation and lung involvement. It was revealed that hypertensive patients were 16.9 times more likely to die as compared to non-hypertensive cases. This proves how hypertension is lethal in COVID-19 cases. Hypertension stimulates the immune system to release cytokines, leading to excessive inflammation, which can cause vascular dysfunction, tissue damage, shock, or multiple organ dysfunction syndrome (MODS). As a result, high blood pressure can increase the risk of organ damage, including heart failure, in patients with severe cases of COVID-19.

Researchers believe that COVID-19-related complications can cause myocardial injury and, ultimately, death. There are several mechanisms reported to analyze the relationship between COVID-19 and CVD. Among cardiovascular diseases, the most frequently reported condition is acute cardiac injury in patients with positive COVID-19 [21,22]. Consequentially, as per our data, of the total 600 cases, 8.8% (n=53) had cardiovascular complications, and among them, 26% (n=47) were death cases. Consecutively, evidence can be seen for the attribution of cardiovascular problems to COVID-19 mortality. It was also indicated that the prevalence of blood clotting among the recovered cases was lower as compared to the mortal cases. Additionally, the present study shows that there is a strong and significant association between death and body mass index obesity in COVID-19 hospitalized patients.

The effect of cardiovascular diseases might be the direct result of the cardiovascular condition itself or attributed to other comorbidities along with a cardiovascular condition [6,23]. Therefore, the main purpose of this study is to show the extent to which the various non-communicable diseases, i.e., diabetes, hypertension, cardiovascular complications, and body mass index, exert their dreadful, life-threatening impact on COVID-19 patients. The population that is at higher risk of developing complications in COVID-19, as previously happened in SARS and MERS, developed severe complications, and ultimately the mortality rate was higher among chronic illness patients. In the United States, 34.2 million people, or 10.5% of the population, have developed DM, whereas 26.8% were diabetics who had COVID-19 [24]. In obese and diabetic individuals, adipose tissue (AT) is impaired and can engage with SARS-CoV-2 both directly and indirectly in various ways. When it comes to direct interactions, AT, which shows higher levels of ACE2 expression (notably in visceral fat), can act as a significant reservoir for the virus, even more than the lungs [22]. Our results clearly demonstrate visible abnormal changes in chest X-rays of patients with multiple comorbidities, with extensive diffuse opacities in the lungs, indicating severe infection and substantial lung damage. This underscores the compounded risk and severity of COVID-19 in patients with multiple health issues, leading to more pronounced respiratory complications and a higher likelihood of severe outcomes.

From the various studies, it can be estimated that diabetic patients, along with being more susceptible to infection, have a poorer prognosis if they get infected with a virus as compared to non-diabetic patients. The worst outcome between the association of viral infection and increased blood glucose levels is not surprising because physiologically increased inflammation rate and morbidity are detrimental effects of increased blood glucose levels [20]. This can be practically seen in our results, which highlight how diabetes proves to be a mortal factor with various peak blood glucose levels. The chest X-ray findings of our study clearly indicate that among diabetic COVID-19 patients, the lungs exhibit hazy opacities, suggestive of moderate infection, highlighting how diabetes can exacerbate COVID-19's effects due to impaired immune response. Moreover, patients with a diabetic history were 33.4 times more likely to die as compared to non-diabetic COVID-19 patients. Since SARS-CoV-2 has the potential to harm the islet of Langerhans and cause the glycosylation of angiotensin-converting enzyme-2 (ACE-2) receptors, it hinders insulin production. This causes an increase in blood glucose, making diabetes more vulnerable to death among COVID-19 patients [22].

This study has a few limitations. Being single-centered and retrospective, its findings may have less generalizability. The reliance on pre-existing medical records may introduce reporting bias. Moreover, selection bias may affect the results of the population due to its single-centered data and convenient sampling technique. Future studies should include a multicentered, prospective approach to offer an additional understanding of the impact of the various comorbidities on COVID-19 patients.

Despite its single-centered approach, the study significantly contributes to understanding the interplay between chronic non-communicable diseases and COVID-19, aiding in the development of better management and intervention protocols for affected patients. The use of chest X-rays allows for an objective and direct visual assessment of lung involvement, offering clear evidence of how these comorbidities affect disease progression.

## Conclusions

It can be concluded that COVID-19-infected hospitalized patients with a history of long-term non-communicable diseases such as diabetes, hypertension, cardiovascular disease, and obesity exhibit prominent changes in chest X-rays with severe lung involvement compared to those without underlying conditions. Moreover, these patients face a greater likelihood of mortality. Therefore, a more focused, vigilant, and tailored treatment approach is needed for the vulnerable population. Additionally, prioritizing the COVID-19 vaccination can help reduce their risk of severe outcomes.
